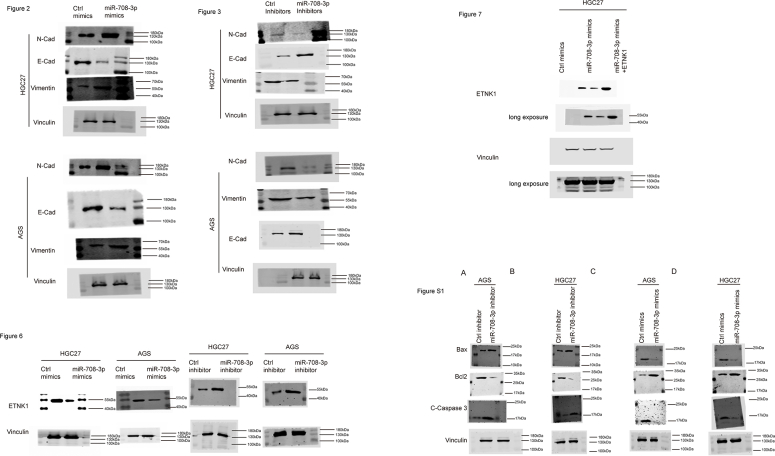# miR-708–3p promotes gastric cancer progression through downregulating ETNK1

**DOI:** 10.1016/j.heliyon.2023.e19544

**Published:** 2023-08-28

**Authors:** Jincai Shang, Qingdong Wang, Jingren Wang, Bo Xu, Shuang Liu

**Affiliations:** Key Laboratory of Microecology-immune Regulatory Network and Related Diseases, School of Basic Medicine, Jiamusi University, Jiamusi, Heilongjiang, 154000, China

**Keywords:** miR-708–3p, ETNK1, Gastric cancer

## Abstract

MicroRNAs (miRNAs) are small, evolutionarily conserved, non-coding RNAs playing a role in the proliferation, metastasis, apoptosis, chemo-sensitivity, and chemo-resistance of gastric cancer, as well as the stemness of gastric cancer stem cells. miR-708–3p induces gastric cancer cell chemo-resistance, but its actual role in gastric cancer progression remains unclear. This paper shows that miR-708–3p is upregulated in gastric cancer samples and that a high miR-708–3p expression in gastric cancer patients is associated with poor overall survival. Our functional study results indicate that miR-708–3p overexpression promotes gastric cancer cell proliferation and migration, inhibits cell apoptosis, and facilitates the transition from the G0/G1 to the G2/M phase. Furthermore, reducing miR-708–3p levels yielded opposite effects. Next, our *in vivo* experiments revealed that miR-708–3p advanced gastric cancer cell growth in nude mice. The underlying mechanism was the regulation of ethanolamine kinase 1 (ETNK1) expression by miR-708–3p, which bound to the 3′UTR of the ETNK1 gene in gastric cancer cells. Finally, the recovery assay results showed that ETNK1 overexpression could slow miR-708-3p-induced gastric cancer progression. In conclusion, we identified a new miR-708–3p/ETNK1 pathway involved in gastric cancer progression. These results may offer new targets for gastric cancer therapy and markers for gastric cancer prognosis.

## 1 Introduction

Gastric cancer (GC) is one of the most prevalent malignant tumors of the digestive tract worldwide. The World Health Organization (WHO) classifies gastric carcinomas by histologic types (tubular, poorly cohesive, mucinous, papillary, and mixed) [1]. According to records, gastric cancer ranked fifth for incidence and fourth for mortality in the world, with one million new cases and about 769,000 deaths in 2020 [2]. In China, it is the second most frequent cancer and the second major cause of cancer-associated deaths, with a high mortality/incidence ratio (0.845) and 5‐year epidemicity (27.6/100,000) [3]. Moreover, 44.21% of global gastric cancer cases occurred in China [4]. Common gastric cancer risk factors include salty diets, smoking, *Helicobacter pylori* infection, and susceptibility to hereditary gastric cancer syndrome [5]. Currently, endoscopic resection is the major treatment for early gastric cancer, while advanced gastric cancer is treated with sequential chemotherapy sessions, beginning with a platinum and fluoropyrimidine combination. Targeted therapies include trastuzumab, ramucirumab, and nivolumab or pembrolizumab [6].

MicroRNAs (miRNAs) have a broad range of fundamental biological activities [7]. They negatively regulate gene expression and can have therapeutic applications [8]. Many microRNAs participate in the development and metastasis formation of gastric cancers. For instance, miR-216 b controls gastric cancer propagation and metastasis by targeting PARK7 [9]. miR-124–3p controls gastric cancer progression and metastasis formation by targeting ITGB3 [10]. Meanwhile, miR-223–3p promotes cell propagation and gastric cancer progression by targeting Arid1a [11]. miR-17–5p drives cellular propagation and gastric cancer progression by targeting RUNX3 [12]. Thus, miRNAs are important markers for gastric cancer diagnosis and prognosis [13]. For example, quantifying miR-195–5p expression after chemotherapy is useful for the prognosis of patients with advanced gastric cancer [14].

miR-708–3p is greatly conserved in distinct species [15]. Cao et al. discovered that it sensitized chemoresistant gastric cancer cells by targeting glycoprotein hormone α polypeptide [16]. However, the influence of miR-708–3p on gastric cancer cell proliferation and migration remains unclear. This paper documents the roles of miR-708–3p in gastric cancer progression and its possible mechanism to offer new therapeutic targets for gastric cancer therapy.

## 2 Materials and methods

### 2.1 Samples from patients and The Genome Cancer Atlas

A total of 34 tumor tissues and 24 paracancerous tissues from Jiamusi University were collected, including 9 pairs of tumor tissues and matching nearby normal tissues. The Ethics Committee of the Jiamusi University approved this study. All patients involved provided informed consent. We stored the samples in RNA later reagent (Ambion, Austin, TX, USA) at −80 °C.

We obtained miRNA information for 372 gastric cancer tissues and 32 nearby non-tumor tissues from The Genome Cancer Atlas (TCGA, https://portal.gdc.cancer.gov). The miR-708–3p expression was evaluated. We grouped the samples by average miR-708–3p expression level (high or low) and used the R software survival package to plot Kaplan–Meier survival curves. Finally, we performed a regression analysis to assess the association between miR-708–3p and ETNK1 expressions.

### 2.2 Cell culture and transfection

The China Infrastructure of Cell Line Resources provided AGS and HGC27 cells. We cultivated the cells at 37 °C in a 5% CO_2_ incubator with Dulbecco's modified Eagle's medium (DMEM) containing 10% fetal bovine serum, nonessential amino acids, glutamine, and antibiotics.

Guangzhou RiboBio provided synthetic mimics of miR-708–3p and a negative control. The miR-708–3p inhibitor and control inhibitor came from GenePharma. The corresponding cDNA fragment (NM_018638.5) was subcloned into a pcDNA3.1 vector (Promega) to construct an expression vector encoding ETNK1.

### 2.3 Cell viability measurement

We seeded the cells in a 96-well plate. After 24, 48, 72, and 96 h of incubation, we washed the cells with a phosphate-buffered solution (PBS). We assessed cell viability using a Cell Counting Kit-8 (Beyotime). Briefly, we mixed the cell-counting solution and DMEM (1:10) and added the mixture into each well. We then incubated the samples at 37 °C for 2 h. Finally, we read the absorbance at 450 nm on a Thermo Fisher Scientific Varioskan Flash multimode reader.

### 2.4 Cell migration assay

We assessed cell migration by performing a transwell assay. We cultured transfected cells for 48 h, then seeded the cells in serum-free medium in the upper wells of the migration chambers (1 × 10^4^ cells/well). The lower wells contained the same medium with 10% serum. After 24 h, we fixed the cells in the lower wells with 2.5% glutaraldehyde, stained them with 0.1% crystal violet, dried them, and counted them.

### 2.5 Cell scratch test

We cultured transfected cells for 12 h, then performed a 2 mm wide scratch along the middle of the culture plate with a 1 mL pipette head. We then washed the culture plate with PBS and checked that no cells remained in the scratches by observation under a microscope. Next, we added culture medium and incubated for 24 h. We then sucked out the culture medium, added 1 mL of 4% paraformaldehyde into each plate, waited 30 min, and took photos under an optical microscope.

### 2.6 Apoptosis analysis

We collected cells, resuspended them in staining buffer, and used an Annexin V–FITC Early Apoptosis Detection Kit (Cell Signaling Technology) according to the manufacturer's instructions. Next, we sorted the cells by flow cytometry and analyzed the data using EXPO32 ADC software (Beckman Coulter). We considered that Annexin V–FITC positive and propidium iodide negative cells were undergoing apoptosis.

### 2.7 Cell cycle analysis

First, we transfected gastric cancer cells for 72 h, then collected and digested them. Next, we fixed the cells with ethanol (75%) at 4 °C for 4 h. We then discarded the supernatant and incubated the cells with an RNA enzyme and iodide (PI, 40%, Sigma-Aldrich). After washing the cells three times with PBS, we performed flow cytometry using a FACSCalibur (BD Biosciences, USA) and analyzed the cell cycle with FACS Diva (BD Biosciences, USA).

### 2.8 Animal experiments

For the *in vivo* tumorigenesis experiments, the Beijing Vital River Laboratory Animal Technology (Beijing, China) provided female athymic BALB/c nude mice aged 6–8 weeks, which we divided into two groups. We housed them in a pathogen-free environment at the Experimental Animal Center of Jiamusi University, under a 12 h light/12 h dark cycle with a standard chow diet. The pLenti–III–miR-GPF tagged vectors were used to generate pLenti-miR-708–3p and lenti-miR-control (empty vector) with puromycin resistance to infect AGS cells and generate stable miR-708–3p. Mice received subcutaneous injections of a suspension of AGS cells (1 × 10^7^ cells/100 μL) transfected with pLenti-miR-708–3p and pLenti-miR-control (empty vector) into the posterior flank. After 5 weeks of treatment, we sacrificed the mice according to the ethical guidelines of the institution. We then measured and compared the tumors of the two groups. We calculated tumor volumes as follows: maximum tumor diameter (L) × right-angle diameter to that axis (W)^2^/2. The animal experiments were approved by the Ethics Committee of Jiamusi University, and were performed according to the animal guidelines of Jiamusi University.

### 2.9 RNA extraction and RT-qPCR

We extracted total RNA from cells using the TRIzol reagent (Invitrogen). For cDNA synthesis and qPCR, we used the PrimeScript RT Reagent Kit (TaKaRa) and the SYBR Premix Ex Taq Kit (TaKaRa), respectively. We measured fluorescence and calculated CT values using a LightCycler 480 system (Roche). The PCR primer sequences were: ETNK1, forward, 5′-GTGCCCAAGCTGAACGTCA-3′, reverse, 5′-TCACCAGGACTACATCCTCCA-3’; β-actin, 5′-CATGTACGTTGCTATCCAGGC-3′, reverse, 5′-CTCCTTAATGTCACGCACGAT-3’. Tianyi Huiyuan (Guangzhou, China) synthesized the primers. We normalized the ETNK1 expression relatively to β-actin expression using the 2^−ΔΔCt^ method.

### 2.10 Western blot analysis

After 10% sodium dodecylsulfate–polyacrylamide gel electrophoresis, we transferred the total protein lysate samples containing 40 μg protein onto polyvinylidene difluoride membranes. Then, we blocked the membranes with 5% non-fat milk in TBST (50 mm Tris-HCl (pH 7.6), 150 mm NaCl, 0.1% Tween-20) at room temperature for 1.5 h. We then washed the membranes three times with TBST buffer and incubated them overnight at 4 °C with anti-ETNK1 (MA5-22149, Thermo Fisher, 1:1000), anti-N-Cad (ab76057, Abcam, 1:1000), anti-Vimentin (ab137321, Abcam, 1:1000), anti-Vinculin (ab130007, Abcam, 1:1000). After washing the membranes with TBST, we incubated them for 1.5 h at room temperature with horseradish peroxidase-labeled IgG. Finally, we washed them again with TBST, treated them with an enhanced chemiluminescence reagent, and revealed them.

### 2.11 Luciferase reporter assay

For 3′-UTR luciferase reporting experiments, we performed subcloning of wild-type and mutant structures of the 3′-UTR of ETNK1 into a pGL3-Basic vector (Promega). Next, we co-transfected cells with the pGL3-Basic vector, pGL3- ETNK1 3′-UTR wild-type or mutant plasmids and miRNA mimics (miR-708–3p mimics or negative control mimics). We then measured firefly and Renilla luciferase activities using a Dual-Luciferase Reporter Assay System (Promega). We reported the firefly luciferase activity normalized relative to Renilla activity as “relative luciferase activity.”

### 2.12 Statistical analysis

We performed all statistical analyses using SPSS 17. We obtained all data from at least three independent experiments and presented the results as the mean ± SD. We compared the two groups using Student's t-test. We considered p-values <0.05 as statistically significant.

## 3 Results

### 3.1 miR-708–3p is upregulated in gastric cancer samples

**Fig. 1:**
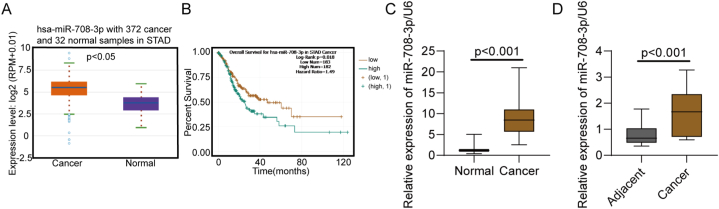
miR-708–3p was upregulated in gastric cancer samples. (A) miR-708–3p expression was markedly lower in 372 gastric cancer samples than in 32 normal samples from TCGA. (B) Overall survival time of gastric cancer patients with low or high miR-708–3p expression (data from the STAD dataset from TCGA). (C, D) Gastric cancer tissues had greatly lower miR-708–3p expression levels than normal or matched nearby tissues (data from our patients).

To document the influence of miR-708–3p on gastric cancer, we analyzed miR-708–3p expression in gastric cancer samples from TCGA and patients. In the STAD dataset from TCGA, the 372 gastric cancer samples had markedly higher miR-708–3p expression levels than the 32 normal samples (Fig. 1A). In gastric cancer patients, a high miR-708-p expression was associated with a worse overall survival rate than a low miR-708–3p expression (Fig. 1B). Furthermore, the 25 gastric cancer tissue samples from our patients had a significantly higher miR-708–3p expression than 15 normal tissue samples (Fig. 1C). In 9 paired tumor tissues, the expression of miR-708–3p was also upregulated than that in paracancerous tissues (Fig. 1D). In conclusion, miR-708–3p expression was greatly upregulated in gastric cancer patients and associated with a lower overall survival rate.

### 3.2 Overexpressing miR-708–3p promotes propagation and migration in gastric cancer cells

**Fig. 2:**
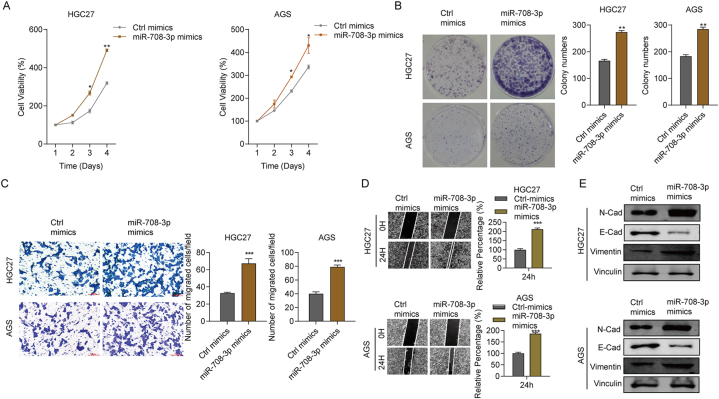
miR-708–3p overexpression promotes the propagation and migration of gastric cancer cells. In both AGS and HGC27 cells, miR-708–3p overexpression (A) greatly enhanced cell viability, (B) greatly increased cell colony generation, and (C) notably promoted cell migration. (D) Scratch test results showing the effect of miR-708–3p mimics on the migration ability of AGS and HGC27 cells. (E) The effect of miR-708–3p mimics on protein expression of the cell migration-related proteins N-cad and Vim. *p < 0.05; **p < 0.01; ***p < 0.001.

To assess whether increased miR-708–3p expression took part in gastric cancer cell proliferation and migration, we elevated miR-708–3p expression in two gastric cancer cell lines (AGS and HGC27) using miRNA mimics. The CCK-8 assay results indicated that miR-708–3p mimic-transfected gastric cancer cells had significantly enhanced cell viability compared with control cells (Fig. 2A). The cell colony formation assay outcomes confirmed that miR-708–3p overexpression promoted cell propagation in HGC27 and AGS cells (Fig. 2B). Next, the transwell assay revealed that the miR-708–3p mimic-transfected cells migrated more than control cells (Fig. 2C). We also assessed the migration ability of AGS and HGC27 transfected with miR-708–3p mimics through a cell scratch test. This experiment showed that miR-708–3p upregulation significantly promoted the migration ability of both cell lines (Fig. 2D). Moreover, miR-708–3p mimic-transfected AGS and HGC27 cells expressed higher protein levels of the cell migration-related genes N-Cad and Vim than control cells (Fig. 2E). In conclusion, miR-708–3p upregulation greatly promotes gastric cancer cell proliferation and migration.

### 3.3 Knocking down of miR-708–3p reduces the proliferation and migration of gastric cancer cells

**Fig. 3:**
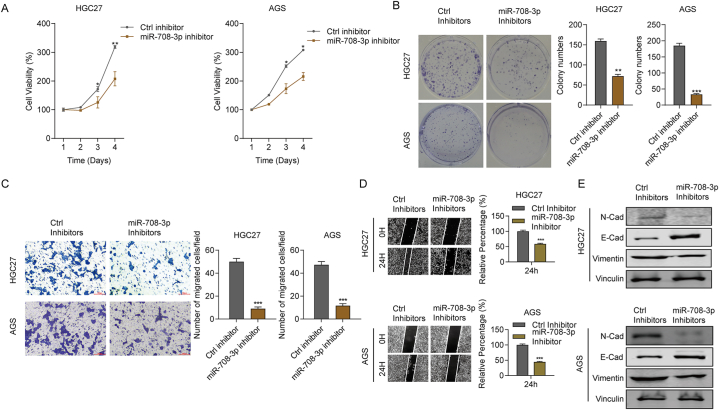
Knocking down miR-708–3p suppresses the proliferation and migration of gastric cancer cells. In both AGS and HGC27 cells, downregulating miR-708–3p (A) greatly reduces cell viability, (B) greatly inhibits cell colony generation, and (C) notably reduces cell migration. (D) Scratch test results showing the effect of a miR-708–3p inhibitor on the migration ability of AGS and HGC27 cells. (E) The effect of miR-708–3p mimics on protein expression of the cell migration-related proteins E-cad, N-cad and Vim. *p < 0.05; **p < 0.01; ***p < 0.001.

Since miR-708–3p overexpression stimulates gastric cancer cell proliferation and migration, we next investigated whether controlling miR-708–3p expression allowed us to control gastric cancer cell progression. CCK-8 and cell colony formation experiment outcomes clearly indicated that controlling miR-708–3p influenced gastric cancer cell proliferation in both AGS and HGC27 cells (Fig. 3A and B). Consistently, the transwell assay (Fig. 3C) and cell scratch test (Fig. 3D) showed that suppressing miR-708–3p inhibited cell migration in both AGS and HGC27 cells. Treating AGS and HGC27 cells with a miR-708-3 inhibitor downregulated the cell migration-related genes N-Cad and Vim，whereas the expression of E-Cad was upregulated in miR-708-3 inhibitor treated AGS and HGC27 cells (Fig. 3E). These results show that reducing miR-708–3p expression may be effective against gastric cancer cell progression.

### 3.4 miR-708–3p regulates gastric cancer cell apoptosis and gastric cancer cell cycle progression

**Fig. 4:**
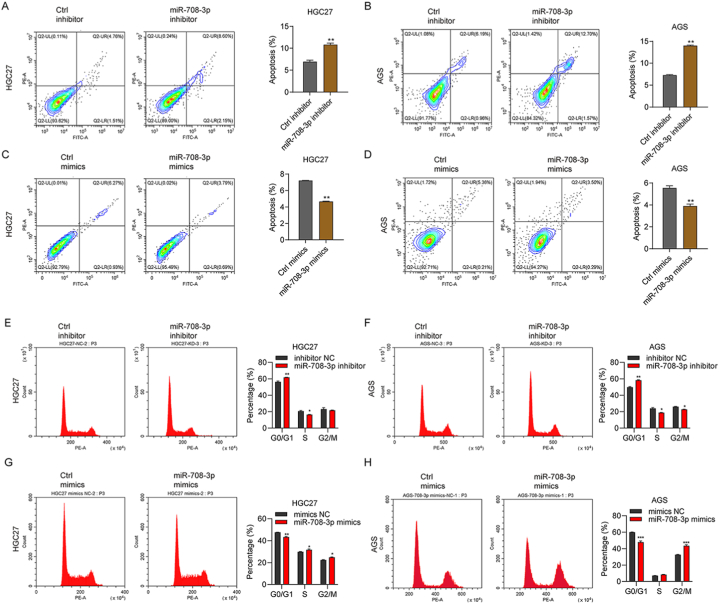
miR-708–3p regulates apoptosis and cell cycle progression. Flow cytometry plots of AGS and HGC27 cells after miR-708–3p inhibition: (A, B) Apoptosis rates were verified by Flow cytometry assay cell in AGS and HGC27 cells after miR-708–3p inhibition; (C, D) Apoptosis rates were verified by Flow cytometry assay cell in AGS and HGC27 cells after miR-708–3p overexpression; (E, F) Cell cycle phases distribution was verified by Flow cytometry assay cell in AGS and HGC27 cells after miR-708–3p knockdown; (G, H) Cell cycle distribution was verified by Flow cytometry assay cell in AGS and HGC27 cells after miR-708–3p overexpression. *p < 0.05; **p < 0.01.

miRNAs take part in DNA degradation and repair, apoptosis control, and cell cycle control [17]. Flow cytometry results showed that inhibiting miR-708–3p significantly increased the apoptosis rate in AGS and HGC27 cells (Fig. 4A and B). However, miR-708–3p mimic transfection yielded contrary results (Fig. 4C and D). Our results also showed that the expression of cleaved caspase-3 (c-caspase-3) and Bax was upregulated in miR-708–3p inhibitor treated AGS and HGC27 cells (Figs. S1A and B), whereas was downregulated in miR-708–3p minics treated AGS and HGC27 cells (Figs. S1C and D). Meanwhile, the anti-apoptotic protein (Bcl-2) was downregulated in miR-708–3p inhibitor treated AGS and HGC27 cells (Figs. S1A and B), whereas was upregulated in miR-708–3p minics treated AGS and HGC27 cells (Figs. S1C and D). Next, we explored the role of miR-708–3p in gastric cancer cell cycle progression. AGS and HGC27 cells transfected with miR-708–3p inhibitor were more in G0/G1 phase and less in G2/M phase compare to that transfected with siCtrl cells (Fig. 4E and F). Conversely, cells transfected with the miR-708–3p mimic were much less in G0/G1 phase and much more in G2/M phase than control cells (Fig. 4G and H). These outcomes indicate that miR-708–3p regulates gastric cancer cell apoptosis and cell cycle progression.

### 3.5 miR-708–3p promotes tumor growth in AGS cells

**Fig. 5:**
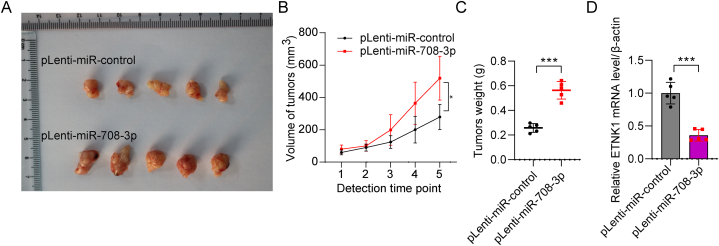
miR-708–3p promotes the growth of AGS cell tumors. (A) Tumors were harvested 35 days after the injection of AGS cells transfected with pLenti-miR-708–3p and pLenti-miR-control. (B) Tumor volumes and (C) tumor weights. *p < 0.05; ***p < 0.001. (D) The expression of intratumor ETNK1 was verified in miR-708–3p over-expression mice tumors using qPCR. ***p < 0.001.

To assess the influence of miR-708–3p on tumor development *in vivo*, we injected pLenti-miR-708–3p and pLenti-miR-control AGS cells in nude mice. miR-708–3p over-expression significantly promoted tumor growth, as indicated by the increase in tumor size (Fig. 5A), volume (Fig. 5B), and weight (Fig. 5C). Our result also showed that the expression of ETNK1 was downregulated in miR-708–3p over-expression mice tumors compared with control using qPCR (Fig. 5D). These results highlight the oncogenic role of miR-708–3p in gastric cancer.

### 3.6 miR-708–3p mediates ETNK1 expression

**Fig. 6:**
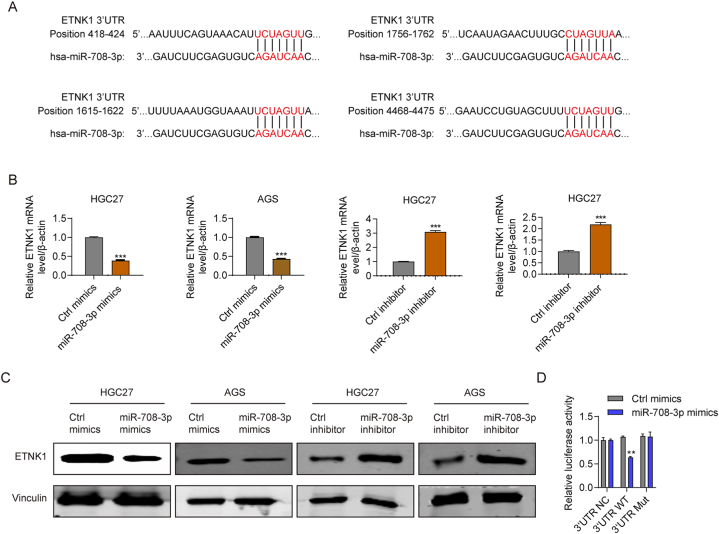
miR-708–3p reduced ETNK1 expression. (A) Sequence alignment of miR-708–3p and the 3′-UTR of ETNK1. (B, C) RT-qPCR and Western blot results showing that miR-708–3p negatively regulated ETNK1 mRNA and protein expression levels in miR-708–3p inhibitor or mimics treated AGS and HGC27 cells. (D) Luciferase activity assay of the 3′-UTR luciferase reporter constructs (wild-type or mutant) with ETNK1 genes. **p < 0.01.

Next, we explored the mechanism of action of miR-708–3p in gastric cancer progression. Using miRNA target forecast methods (targetscan.org and mirdb.org) to identify miR-708–3p target genes revealed ETNK1 as a miR-708–3p tentative target. ETNK1 is an ethanolamine kinase involved in the phosphatidylethanolamine synthesis pathway [18]. According to our target forecast, ETNK1 contains four miR-708-3p-binding sequences (Fig. 6A). To assess the relationship between miR-708–3p and ETNK1 expressions, we quantified ETNK1 expression in miR-708–3p overexpressing AGS and HGC27 cells. As shown in Fig. 6B–C, miR-708–3p overexpressing cells had lower ETNK1 mRNA and protein expression levels than control cells, whereas the expression of ETNK1 was upregulated in miR-708–3p inhibitor treated AGS and HGC27 cells in mRNA levels (Fig. 6B) and protein levels (Fig. 6C). Next, to confirm that the inhibition of expressing target luciferase reporter genes relied on combining their complementary 3′-UTR sequences to the miR-708–3p seed sequence, we inserted a 3-nucleotide mutation to the 3′-UTR of the target genes (Fig. 6A). The wild-type 3′-UTR yielded a significantly lower luciferase activity than the 3′-UTR mutation and control, which had similar activities (Fig. 6D). Altogether, these results show that miR-708–3p negatively modulates ETNK1 expression in gastric cancer cells by directly targeting its 3′-UTR sequence.

### 3.7 miR-708–3p drives proliferation and migration by regulating ETNK1

**Fig. 7:**
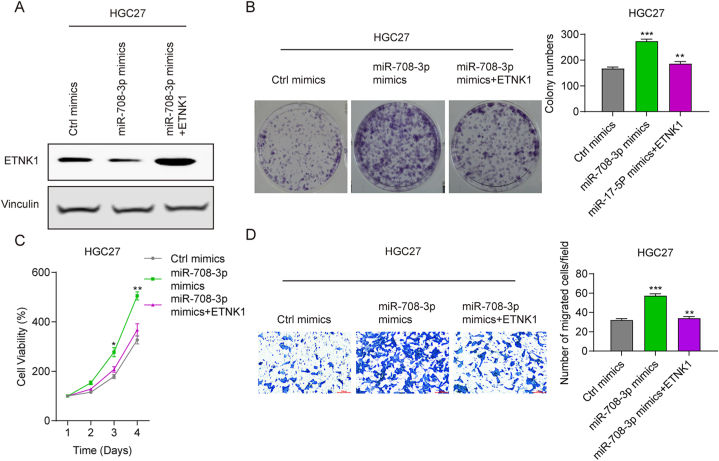
miR-708–3p promotes propagation and migration by regulating ETNK1. (A) ETNK1 expression, (B) cell viability, (C) cell colony formation numbers, and (D) cell migration numbers in HGC27 cells transfected with mimics control, miR-708–3p mimics, or miR-708–3p mimics + ETNK1. *p < 0.05; **p < 0.01; ***p < 0.001.

To find out whether miR-708–3p drives proliferation and migration by regulating ETNK1, we performed cell function tests in AGS and HGC27 cells. Transfecting the miR-708–3p mimic greatly reduced ETNK1 expression, while co-transfecting the miR-708–3p mimic and ETNK1 increased it (Fig. 7A). Meanwhile, the miR-708–3p mimic enhanced cell viability, while the miR-708–3p mimic and ETNK1 markedly inhibited it. The miR-708–3p mimic and ETNK1 co-transfected cells and control cells had similar viability levels (Fig. 7B). Similarly, overexpressing ETNK1 reversed the effect of the miR-708–3p mimic on cell colony formation in HGC27 cells (Fig. 7C). Furthermore, the transwell test revealed that miR-708–3p mimic-transfected HGC27 cells has more migrated cells than control. Meanwhile, HGC27 cells transfected with both miR-708–3p mimic and ETNK1 showed less migrated cells compare to that transfected with miR-708–3p mimic alone (Fig. 7D). Therefore, miR-708–3p promotes gastric cancer cell proliferation and migration by inhibiting ETNK1.

## 4 Discussion

miRNAs are small non-coding regulatory RNAs of 17–25 nucleotides. Nowadays, the miRBase database contains more than 38,589 miRNAs from over 271 organisms. In cancer cells, miRNA expression depends on cancer type, phase, and other clinical variables, making miRNA profiling a useful cancer diagnosis and prognosis method [19]. miRNAs regulate various aspects of gastric cancer, including propagation, metastasis, apoptosis, chemo-sensitivity, chemo-resistance, and cancer stem cell stemness by regulating oncogenes or tumor-suppressive genes [20].

miR-708–3p plays a role in various diseases, including cancers. Wang et al. found that the high miR-708–3p expression in the bone tissue of ovariectomized rats and patients with osteoporosis had a synergistic effect with miR-708–5p in osteoporosis development in different time and space patterns [21]. Liu et al. observed that miR-708–3p aggravates idiopathic pulmonary fibrosis by binding to ADAM17 [15]. Qu et al. revealed that miR-708–3p moderated the inflammation and damage of myocardial cells by targeting ADAM17 [22]. Leidinger et al. revealed that the expression of hsa-miR-708–3p in prostate carcinoma tissues was lower than that in benign prostatic hyperplasia tissues [23]. Lee et al. showed that breast cancer patients with metastasis had markedly lower miR-708–3p expression than patients without metastasis. As a cancer suppressor miRNA, it completely inhibits breast cancer metastasis and chemotherapy resistance by regulating MT (directly targeting EMT activators, such as ZEB1, CDH2, and vimentin) [24].

miRNAs are significant diagnostic markers and therapeutic targets for many diseases [15]. This paper is the first to reveal that miR-708–3p is upregulated in gastric cancer samples from TCGA and clinical samples. Gastric cancer patients with low and high miR-708–13p expression levels had drastically different overall survival rates. miRNAs affect nearly every cancer process, such as propagation, apoptosis, invasion/metastasis, and angiogenesis [19]. Indeed, using miRNA mimics or inhibitors, we found that miR‐708–3p influenced the proliferation and migration of gastric cancer cells. The CCK-8, cell colony formation, transwell, and cell scratch tests all showed that an upregulation of miR-708–3p promoted gastric cancer cell proliferation and migration, whereas downregulation suppressed migration. Additionally, miR-708–3p mimic-transfected AGS and HG27 cells had higher cell viability, cell colony numbers, cell migration numbers, and cell migration-related genes N-cadherin and Vim expression levels than control cells. Meanwhile, transfecting cells with a miR-708–3p inhibitor reduced all these parameters.

Since miRNAs regulate the cell cycle and apoptosis [25], we next determined the influence of miR-708–3p on gastric cancer cell apoptosis and cell cycle progression. Flow cytometry results indicated that upregulating miR-708–3p reduced gastric cancer cell apoptosis, whereas downregulating it induced gastric cancer cell apoptosis in both AGS and HGC27 cells. Furthermore, AGS and HGC27 cells transfected with miR-340–5p mimics showed cell cycle arrest at the G0/G1 phase, and GC cells transfected with miR-340–5p inhibitor promoted cell cycle progression. Next, to confirm these *in vitro* results *in vivo*, we established a gastric cancer animal model using AGS cells stably expressing miR‐708–3p. Using this model, we showed that miR-708–3p overexpression increased tumor size, volume, and weight.

miRNAs post-transcriptionally repress gene expression by recognizing complementary target sites in the 3′-UTR of target mRNAs [19]. Using miRNA target forecast methods (targetscan.org and mirdb.org) to find miR‐708–3p target genes in gastric cancer progression, we identified ETNK1. It encodes an ethanolamine kinase which plays roles in the first committed step of the phosphatidylethanolamine synthesis pathway. This cytosolic enzyme is specific to ethanolamine, and its kinase activity towards choline is negligible [18]. A study showed that ETNK1 washighly expressed in the stomach using RNA-seq from 27 tissues from 95 humans [26]. ETNK1 is a chemosensitivity-related gene in gastric cancer cell lines [27]. miRNAs improve the response of laryngeal and lingual squamous cell carcinoma cells to platinum chemotherapy by regulating several metabolic factors and enzymes, involving ETNK1 [28]. Li et al. reported that miR-199a-3p promotes aggression and migration of gastric cancer cells by targeting ETNK1 [18].

Luciferase assay results showed that miR‐708–3p regulated the mRNA and protein levels of ETNK1 by binding the target gene's 3′UTR. We found a negative association between miR‐708–3p and ETNK1 expressions using 372 samples from the STAD dataset. Finally, a recovery assay confirmed that miR‐708–3p promoted gastric cancer cell progression by regulating ETNK1.

In conclusion, we found a new miR-708–3p/ETNK1 pathway, which is crucial for gastric cancer progression and may be a new marker or target for gastric cancer therapy.

## Author contribution statement

Shuang Liu: Conceived and designed the experiments; Wrote the paper. Jincai Shang: Conceived and designed the experiments; Performed the experiments; Analyzed and interpreted the data; Wrote the paper. Qingdong Wang: Performed the experiments; Analyzed and interpreted the data. Jingren Wang; Bo Xu: Analyzed and interpreted the data; Contributed reagents, materials, analysis tools or data.

## Data availability statement

Data included in article/supp. material/referenced in article.

## Declaration of competing interest

The authors declare that they have no known competing financial interests or personal relationships that could have appeared to influence the work reported in this paper.

## Figures and Tables

**figs1:**