# Supplementary material for: miR-708–3p promotes gastric cancer progression through downregulating ETNK1
Source: Heliyon. 2023 Aug 28;9(9):e19544. doi: 10.1016/j.heliyon.2023.e19544 (PMC10558739; doi:10.1016/j.heliyon.2023.e19544)
Supplement: Multimedia component 1 [file mmc1.docx]

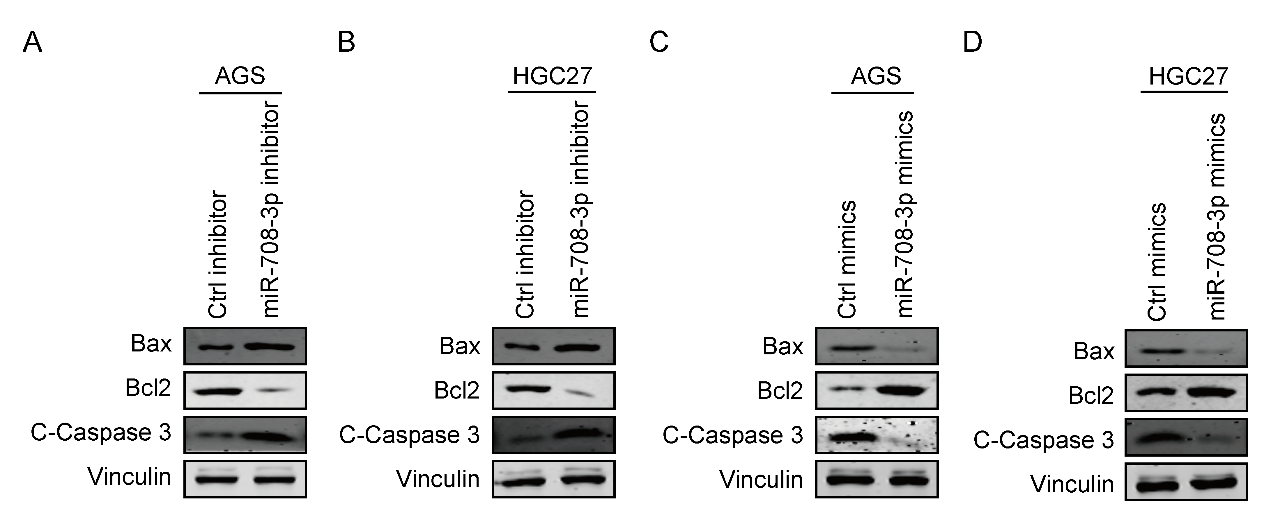


Figure S1. miR-708-3p suppresses apoptosis in gastric cancer. Western blotting analysis of Bcl2, Bax and cleaved-Caspase3 (c-Caspase3) in AGS and HGC27 cells with inhibitor (A, B) or minics (C, D) of miR-708-3p.
